# Histone Deacetylase Inhibitor Treatment Increases Coronary t-PA Release in a Porcine Ischemia Model

**DOI:** 10.1371/journal.pone.0097260

**Published:** 2014-05-12

**Authors:** Kristina Svennerholm, Niklas Bergh, Pia Larsson, Sverker Jern, Göran Johansson, Björn Biber, Michael Haney

**Affiliations:** 1 Anesthesiology and Intensive Care Medicine, Institute of Clinical Science, Sahlgrenska Academy, Gothenburg University, Gothenburg, Sweden; 2 The Wallenberg Laboratory for Cardiovascular Research, Institute of Medicine, Sahlgrenska Academy, Gothenburg University, Gothenburg, Sweden; 3 Anesthesiology and Intensive Care Medicine, Institute for Surgical and Perioperative Science, Umeå University, Umeå, Sweden; Scuola Superiore Sant'Anna, Italy

## Abstract

**Background:**

The expression of the tissue plasminogen activator gene can be affected by histone deacetylation inhibition and thus appears to be under epigenetic control.

**Objectives:**

The study aimed to test if *in vivo* pharmacological intervention by valproic acid treatment would lead to increase in tissue plasminogen activator release capacity.

**Methods:**

In an anaesthetized pig model, a controlled transient coronary occlusion was used to stimulate coronary tissue plasminogen activator release in a valproic acid treated (one week) and a non-treated group. Coronary venous blood samples from the ischemic region were collected, great cardiac vein thermodilution flow measurements were performed, and trans-coronary tissue plasminogen activator fluxes were calculated. Plasminogen activator inhibitor-1 was also measured.

**Results:**

Adequate sampling from the affected area after the 10 minute ischemic period was confirmed by lactate measurements. Fluxes for tissue plasminogen activator at minutes 1, 3, 5, 7 and 10 were measured and then used to present cumulative net tissue plasminogen activator release for the whole measurement period for both groups. Area under the curve was higher for the valproic acid treated group at 10 minutes; 932±173 nanograms (n = 12) compared to the non-treated group, 451±78 nanograms (n = 10, p = 0.023). There was no difference in levels of plasminogen activator inhibitor-1 between groups.

**Conclusions:**

These findings support a proof of concept for histone deacetylation inhibition positive effect on tissue plasminogen activator expression in an in vivo setting. Further studies are needed to find an optimal way to implement histone deacetylation inhibition to achieve desired clinical changes in tissue plasminogen activator expression.

## Introduction

Myocardial infarction is most often caused by intravascular thrombus formation, usually at an atherosclerotic lesion. The clinical outcome of the thrombotic event can depend on whether or not the thrombus is rapidly removed by the endogenous fibrinolytic system, or if it persists leading to prolonged regional ischemia and irreversible tissue damage. Healthy vascular endothelium produces and stores the key fibrinolytic enzyme, tissue-type plasminogen activator (t-PA). This t-PA is quickly released in the vicinity of the clot when a clotting process is initiated. Factors stimulating acute release of t-PA in a thrombotic situation include coagulation activation products, adrenergic influence, and ischemia [1]–[7].

Endothelial-derived t-PA rapidly and locally activates fibrin degradation, which resolves the thrombus allowing reperfusion to take place. Adequate endogenous fibrinolytic capacity and activity thus provides protection from thrombotic complications. A number of clinical conditions are associated with a reduced capacity for acute t-PA release, including hypertension, chronic renal failure, obesity and coronary atherosclerosis [8]–[12]. These are also well-established risk factors for myocardial infarction. The same phenomenon has been shown among smokers [13] and in patients with elevated CRP levels [14]. Furthermore, there are clinically relevant t-PA polymorphisms in the general population, and individuals with genetically impaired t-PA release have increased risk for cardiovascular thrombotic events [15]–[17].

There have long been efforts to find a pharmacological means to increase the endogenous capacity of the fibrinolytic system, and this potentially could be a novel means to prevent thrombotic conditions. This may be achieved by increasing the endothelial pools of t-PA or by decreasing the circulating plasma levels of plasminogen activator inhibitor-1 (PAI-1). Administration of recombinant t-PA has been used as an acute treatment alternative for patients presenting an acute atherothrombotic event for almost three decades [18]. However, optimal endogenous fibrinolytic capacity is more desirable since it is localized and immediate [19], [20]. Systemic thrombolysis confers risk for bleeding complications and the efficacy of the treatment is limited by its late onset. Compared to existing thrombolytic therapy, stimulation of endogenous t-PA production would likely have several benefits. By priming the endothelium to produce more t-PA protein and restore the capacity for t-PA release, t-PA levels will be increased only locally around the clot, which might reduce the bleeding risk associated with systemic administration of recombinant t-PA.

Up until now, no clinically relevant means to stimulate the endogenous fibrinolytic system in man has been described. Our group and others have recently demonstrated that the expression of the t-PA gene is dramatically induced by substances affecting histone acetylation, histones that are in complex with DNA in the chromosomes and thus appears to be under epigenetic control [21]–[24]. Increased acetylation of histones occurs through blocking of the enzyme histone deacetylase with histone deacetylase inhibitors (HDACi) [25], [26].

This effect has been demonstrated with the clinically used anticonvulsant and mood-stabilizing drug valproic acid (VPA). Increase in t-PA production after HDAC inhibition by VPA has been demonstrated in vitro in human endothelial cells, but has so far not been demonstrated in vivo [23]. Therefore, the aim of this study was to test if *in vivo* pharmacological epigenetic intervention, by VPA treatment, would lead to an increase in t-PA release capacity in the pig heart when using a clinically relevant endothelial stimulus, ischemia. We aimed to test this concerning constitutive (resting) t-PA release as well as facilitative (stimulated) t-PA release.

## Materials and Methods

This study was performed after approval from the Regional Animal Research Ethics Committee in Umeå, Sweden (document A 123-10), and was conducted in line with the ‘Humane Care and Use of Laboratory Animals and the Guide for the Care and Use of Laboratory Animals’ (1996), from the National Academy of Sciences' Institute for Laboratory Animal Research, USA.

The study was performed using domestic land-race pigs, which were raised and supplied for research purposes by a local school of agriculture. The animals were fasted for 12 hours with free access of water prior to the experiment. The pigs in the intervention group were brought to lab and kept in a large pen for 1 week before the trial. This was for twice-a-day administration of valproic acid 500 mg (Ergenyl Retard, Bromma, Sweden) with food, up to and including the afternoon on the day before the experiment. There were approximately 12 hours between the last VPA dose and plasma sampling for VPA concentration.

### Preparation

After premedication with ketamine 10 mg·kg^−1^ (Ketalar, Pfizer, Morris Plains, New Jersey, USA) and xylazine 2 mg·kg^−1^ (Rompum vet, Bayer AB, Lyngby, Denmark) intramuscularly, an ear vein was cannulated for anesthesia induction with pentobarbital 10 mg·kg^−1^ (Pentobarbitalnatrium, Apoteksbolaget, Stockholm, Sweden). The anesthesia was maintained throughout the experiment with a continuous infusion of fentanyl 20 microgram·kg^−1^·h^−1^ (Fentanyl, Braun, Melsungen, Germany), midazolam 0.3 mg·kg^−1^·h^−1^ (Dormicum, Roche, Basel, Switzerland) and pentobarbital 5 mg·kg^−1^·h^−1^. No muscle relaxants were used. The animals were mechanically ventilated (Evita 4, Dräger, Kiel, Germany) in volume-controlled mode with a mixture of 30% oxygen. Ventilation was adjusted to normocapnia by end-tidal capnometry and intermittent arterial blood gases analyses (ABL-5, Radiometer, Denmark). Intravenous Ringer's acetate was infused at 15 ml kg·h^−1^ throughout the protocol. Body temperature was measured rectally and maintained at 38–39°C by a heating pad and cloth covers. At the end of the experiment the animals were euthanized by an anesthesic overdose, a bolus injection of intravenous pentobarbital 60 mg/ml (3–4 ml), along with potassium chloride 40 mmol.

**Figure 1:**
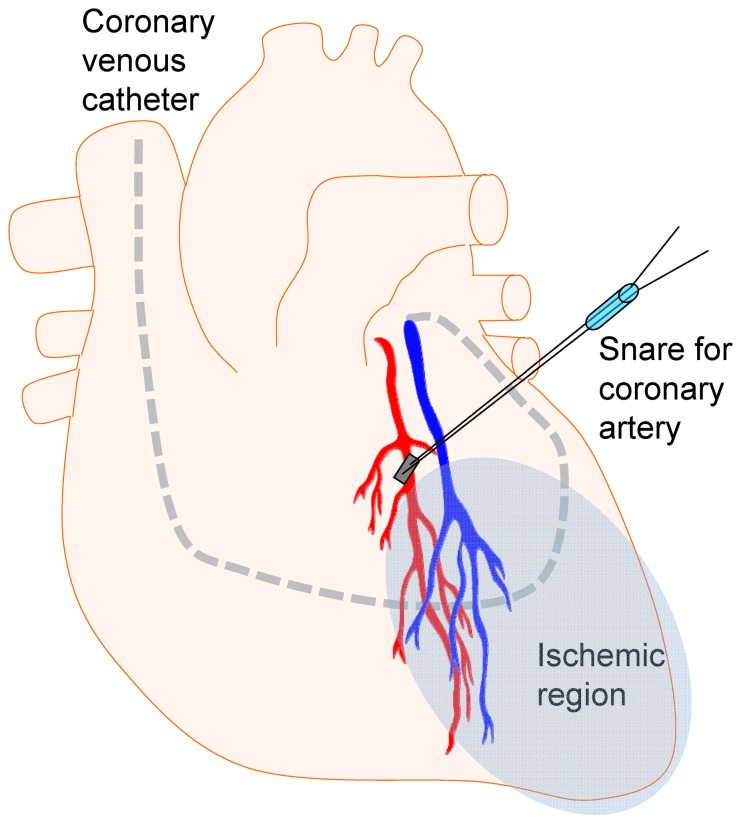
Schematic image of the in vivo heart preparation. The left anterior descending artery and corresponding vein are depicted. A coronary snare is placed in the mid region of this artery, and when drawn, this generates a region of ischemia corresponding to a larger part of the anterior left ventricle. The coronary venous catheter is introduced through the right atrium, coronary sinus, and finally placed with the sampling tip in the proximal great cardiac vein, draining the left anterior descending vascular region (ischemic area).

The animals were placed in the dorsal position after induction of anesthesia. Tracheostomy with an endotracheal tube inserted was performed. Neck artery and veins were exposed through right- and left-sided neck dissection. Through an internal jugular vein a triple-lumen central venous catheter (Arrow-Howe Multi-Lumen Central Venous Catheter, Vingmed, Järfälla, Sweden) was inserted to measure right atrial pressure (CVP) and for administration of fluids and drugs. An arterial catheter was introduced through a cutdown on a branch of the carotid artery, and was used for invasive blood pressure measurement, sampling of aortic blood and intermittent blood gases. In order to sample blood from coronary veins and quantify coronary venous blood flow, an 8F dual-thermistor, ball-tipped coronary sinus (CS) catheter (CCS-7 U-90A; Webster Labs, Altadena, California, USA) was inserted through the right external jugular vein and placed with the sampling and infusion tip in the great cardiac vein (GcV) using fluroscopic guidance. Correct position was confirmed with measurement of venous oxygen saturation from GcV, as well as measurements of GcV flow (Q_GCV_), which was measured using retrograde thermodilution [27]. This position meant that the blood sampling (and thermodilution injecting) distal port on the catheter lay very close to the coronary vein from the left anterior descending artery (LAD) region, and that the external thermistor on the CS catheter lay proximal to the hemiazygous entry to the common distal coronary sinus. This allowed sampling of GcV blood and Q_GCV_ undisturbed by contact from central venous blood. Impedance signals were processed by a two-channel Wheatstone bridge (CBA-210, Webster Lab, Altadena California, USA) and recorded digitally on a multi-channel amplifier (AcqKnowledge, Goleta, California, USA). Midline sternotomy was performed followed by pericardiotomy. A patched snare was placed around the middle portion of the LAD artery. Care was taken not to occlude or injure the corresponding vein since this could interfere with coronary venous sampling. A schematic depiction of the heart preparation is shown in Figure 1. At the end of the preparation, heparin 5000 units were administered.

The heart rate was recorded continuously using ECG along with mean arterial pressure (MAP), CVP and rectal temperature. Arterial and coronary venous blood gases were measured before and after cardiac ischemia. All data were saved in digital format using a computer based multi-channel acquisition and analysis system (AcqKnowledge, Biopac, California, USA).

### Protocol

Following the preparation, the animals were allowed to rest under anesthesia for at least 1 hour before baseline blood sampling, which included a single blood sample for measurement of plasminogen activator inhibitor (PAI-1) activity (Porcine PAI-1 ELISA, Innovative Research, Southfield, USA). Acute regional myocardial ischemia was created by a controlled snare-occlusion of the middle part of LAD for 10 minutes. During reperfusion, simultaneous arterial and coronary venous blood samples were obtained at minutes 1, 3, 5, 7 and 10. Serial measurements of Q_GCV_ were collected, one after each blood sampling. Concerning blood collection, the first portions of blood corresponding to the volume of catheters were discarded for each sampling. The 2 ml of blood that were collected for t-PA analysis was put in chilled tubes containing 0.2 ml 0.45 M sodium citrate buffer (Stabilyte, Biopool AB, Umeå, Sweden) and directly placed in an ice bath. Blood samples for quantifying lactate were taken at the same time as those taken for t-PA analyses. Immediately after the 10 minutes sampling period, the samples were centrifuged (for 20 min at 4°C and 2000 G). The plasma was stored at -70°C in five aliquots until being thawed for analysis.

### Laboratory analysis

VPA concentration analyses were performed by standard methods at the Department of Clinical Chemistry at the Sahlgrenska University Hospital, Gothenburg, Sweden (ARCHITECT iValproic Acid assay, Abbott, Abbott Park, Illinois, USA). Lactate from GcV samples was measured (ABL-5, Radiometer, Denmark). Plasma levels of PAI-1 activity were determined by an enzyme-linked immunoassay (Porcine PAI-1 ELISA, Innovative Research, Southfield, USA). Plasma levels of total t-PA antigen were determined by an enzyme-linked immunoassay (TintElize t-PA antigen ELISA, Trinity Biotech, Bray, Ireland). For porcine standard, recombinant porcine t-PA diluted in t-PA depleted porcine plasma was used. Samples from each animal were analysed on one single plate, and all samples were analysed in duplicates with a goal of mean intra-assay coefficient of variation less than 5%.

Net release/uptake of total t-PA across the cardiac vascular bed was calculated from the product of the arterio-venous concentration gradient of t-PA (C_GCV_ – C_A_) and the local plasma flow (Q_GCV_×(1-Hct)), where *C_GCV_* is the venous concentration and *C_A_* is the arterial concentration, along with hematocrit (Hct). Hematocrit was calculated from measured hemaglobin concentration in grams/liter (OSM-3, Radiometer, Copenhagen, Denmark) multiplied by 0.0485.

### Analysis

In order to be included for analysis, it was required that the animal successfully completed the reperfusion period with demonstrated adequate GcV venous sampling of the ischemic region for all 5 sampling points during the immediate 10 minute reperfusion period. More than doubling of trans-coronary lactate flux (positive lactate production) was used to confirm successful ischemia and reperfusion sampling.

Point measurements for t-PA flux at baseline (pre-ischemia) and minutes 1, 3, 5, 7, and 10 were measured, and then used to present cumulative t-PA flux over the measurement period. This was done by using each individual t-PA flux change from baseline, together with time for the measurement, in order to calculate the area under the curve (integral) for cumulative t-PA release.

Data are presented as mean ± standard error of the mean (SEM). The statistical analyses were performed using SPSS (IBM, Armonk, New York, USA). All of the variables were tested and confirmed for normal distribution using the Kolmogorov-Smirnov test. Testing for differences between measurements within groups, as well as between groups for the sequence, was performed using mixed design analysis of variance (ANOVA). Where a single contrast was performed between 2 groups, a t test was used. A p value less than 0.05 was considered significant.

## Results

Twenty-nine animals were initially included, and 22 completed the protocol successfully, with verified (by increase in lactate) coronary venous sampling of myocardial ischemia. There were 12 in the treatment group, and 10 in the non-treatment group. The 7 that did not complete the protocol dropped out because of malignant dysrhythmia and/or circulatory collapse during ischemia or reperfusion. One animal had an anomalous coronary vein anatomy where GcV sampling of the ischemic area was not adequate. The animals had a mean body weight of 41±7 kg (standard deviation). There was no detectable serum VPA in any of the samples, which were taken approximately 12 hours after the last tablet administration.

**Figure 2:**
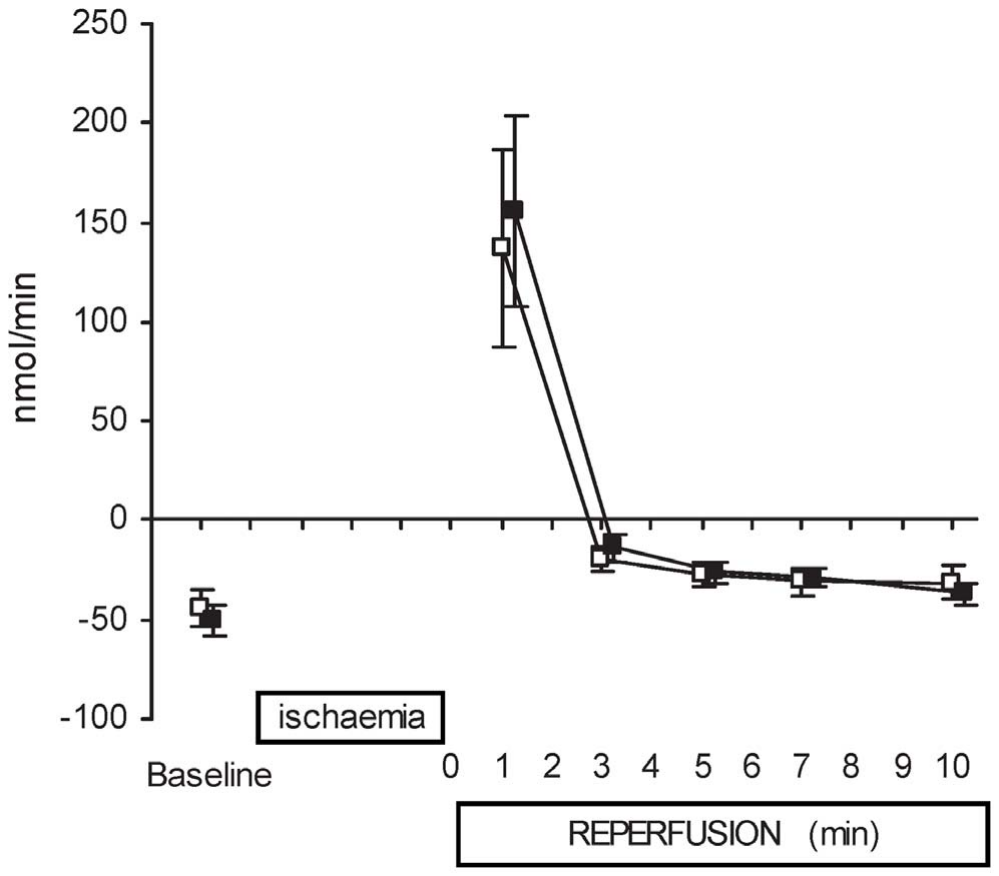
Absolute values of coronary lactate-fluxes. These are shown for pre-ischemia (baseline), after 10 min of ischemia followed by 10 min of reperfusion for treated group (filled squares, n = 12) and for control group (open squares, n = 10). Data are presented as mean ± SEM. Sampling of the ischemic area, as confirmed by positive lactate flux (regional myocardial lactate production in response to the local coronary occlusion), was observed in all animals (not shown individually) and is reflected by the immediate high positive lactate flux noted at reperfusion minute 1. This regional lactate flux was quickly ‘washed out’. No difference was found between groups (p = 0.853, mixed between-within subjects ANOVA).

The hemodynamic events, before ischemia and then during reperfusion, showed that VPA treatment did not affect heart rate, blood pressure, coronary venous flow or lactate production during the baseline or post-ischemic period (Table 1). Coronary ischemia led to a clear increase in coronary venous blood flow (CSF) in both groups, which was highest at minute 1 after snare release, and then progressively returned towards pre-ischemia levels during the 10 minute reperfusion measurement period. Lactate net consumption in the pre-ischemia measurement is observed for both groups, and clear regional myocardial lactate production during regional ischemia was noted in the first reperfusion minute (Figure 2). There was no difference noted between the treatment and non-treatment groups in systemic arterial t-PA levels before or after the ischemic provocation.

**Table 1:** Hemodynamic results.

														Within groups
														ANOVA
	group	Baseline		1 min		3 min		5 min		7 min		10 min		*p* =
Weight (kg)	Control	39.8	±2.8											
Weight (kg)	Treated	42.0	±1.8											
HR	Control	88	±4.6	87	±4.1	88	±4.1	88	±3.7	87	±3.9	88	±3.6	*0.981*
HR	Treated	85	±4.0	79	±3.8	84	±5.6	84	±4.6	80	±3.0	81	±3.0	*0.239*
MAP	Control	87	±2.6	83	±3.7	84	±2.8	84	±3.6	83	±3.5	84	±3.7	*0.376*
MAP	Treated	81	±4.9	73	±5.6	73	±4.7	74	±4.6	77	±4.3	78	±4.2	*0.069*
CSF	Control	75.0	±12.4	117.0	±18.0 *	110.0	±19.1 *	100.0	±18.7 *	80.0	±14.9	80.0	±16.0	*<0.001*
CSF	Treated	65.0	±6.8	123.0	±16.7 *	112.0	±11.4 *	88.0	±5.6 *	72.0	±6.3	63.0	±6.9	*<0.001*

HR =  heart rate (bpm); MAP =  mean arterial pressure (mm Hg); CSF  =  coronary sinus blood flow (mL/min). Control group (n = 10), treated group (n = 12). Data are presented as mean ± 95% SEM. * = p<0.05 using repeated measures ANOVA and when significant was followed by paired t test vs. baseline. A between-groups t test was performed, but no differences were found.

**Figure 3:**
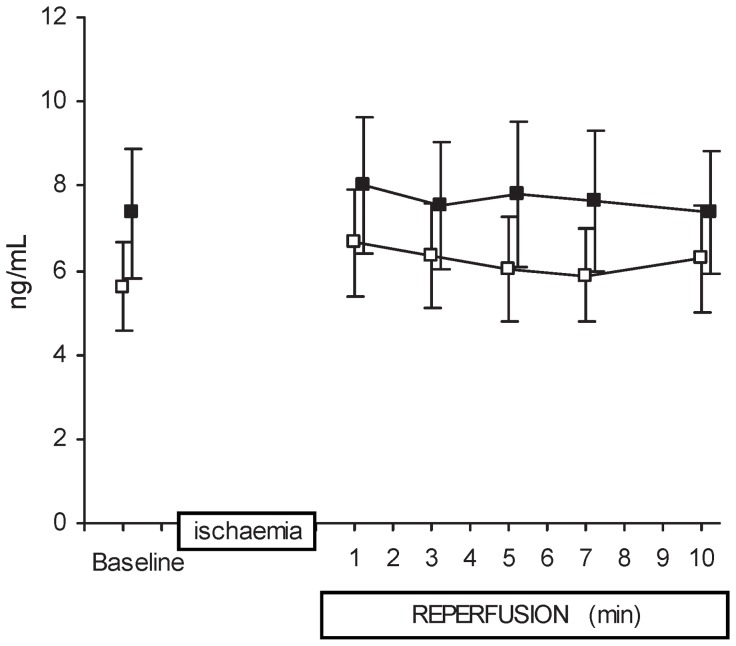
Arterial t-PA-concentrations. Absolute t-PA concentrations in arterial blood are shown for pre-ischemia (baseline), as well as for the period after 10 min of ischemia and 10 min of reperfusion. Both the treated group (filled squares, n = 12) and the control group (open squares, n = 10) are shown. Data are presented as mean ± SEM. No differences were noted within groups over time, or between groups at any of the time intervals (mixed between-within subjects ANOVA). There was no sign here that the valproic acid treatment led to higher systemic blood levels of t-PA in the resting state. These results also showed that the coronary t-PA release did not affect systemic arterial t-PA levels.

**Figure 4:**
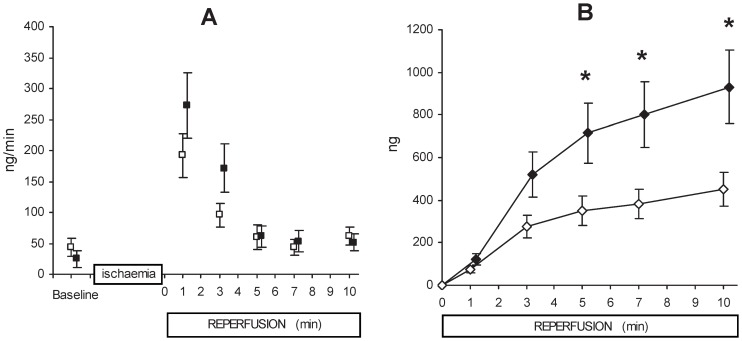
Coronary t-PA-flux. Coronary t-PA fluxes are shown in Panel A for pre-ischemia (baseline), after 10 min of ischemia followed by 10 min of reperfusion for treated group (filled squares, n = 12) and for control group (open squares, n = 10). These are point measurements for observed t-PA fluxes at minutes 1, 3, 5, 7, and 10. One can note that there are different baselines for the two groups. The main result is derived from Panel A, and presented as the cumulative t-PA release over time in Panel B, as area under the curve (AUC). There is a clearly higher cumulative t-PA release for the VPA treated group (mixed between-within subjects ANOVA, p = 0.023); treated group (filled diamonds, n = 12) and control group (open diamonds, n = 10) data presented as mean ± SEM. There were also differences between groups (larger cumulative t-PA release in the treatment group) for specific measurements at minutes 10, 7, and 5 (t-test).

There was no difference between treatment and non-treatment groups in plasma PAI-1 activity (number of measurements n = 12, n = 7, respectively) (17.4±2.9 vs. 18.1±2.2 nanograms/ml respectively). The arterial plasma t-PA concentration reflects t-PA release from all organs and was no different between groups pre-ischemia (baseline), and it remained unchanged over the time-course from pre-ischemia as well as through 10 minutes reperfusion for each group (Figure 3). Coronary ligation and 10 minutes of myocardial ischemia led to positive trans-coronary t-PA release for the immediate ischemic period (Figure 4, Panel A). Absolute t-PA release results at specific times during reperfusion show that the groups did not start at exactly the same baseline, and that they both showed an early larger amount of t-PA release. There was a positive net trans-coronary t-PA release throughout the 10 minute reperfusion period (Figure 4, Panel A). The main findings were that there was a clear positive treatment effect for increased cumulative t-PA release (calculated as AUC) for the whole 10 minutes post-ischemic period for the treatment group (Figure 4, Panel B). Area under the curve was higher for the valproic acid treated group at 10 minutes; 932±173 nanograms (n = 12) compared to the non-treated group, 451±78 nanograms (n = 10, ANOVA mixed between-within subjects, p = 0.023). A higher cumulative t-PA release was evident for the treatment group at 7 and 5 minutes.

## Discussion

The main finding demonstrates for the first time in an *in vivo* system that pharmacological stimulation of the endogenous fibrinolytic system with HDACi increases acute facultative t-PA release. Valproic acid pre-treatment led to increased t-PA release by a standardised acute ischemic provocation in this porcine model. The effect size was measureable even in the quite small groups. Other notable and related findings here include that VPA did not have any effect on basal t-PA or PAI-1 concentrations, suggesting that constitutive release was not affected by VPA treatment.

Previously *in vitro* findings have demonstrated that VPA in lower doses can increase t-PA release, and in higher doses cause manifold increase in t-PA release [23], [24]. The mechanism has been confirmed to be VPA's inhibitory effect on the enzyme histone deacetylase, which leads to induction of t-PA transcription in different cell types [23], [28]. Evidence for this effect has been presented for clinical material; Olesen et al presented in a Danish national registry study an association between antiepileptic treatment with VPA and a 40% reduced risk for myocardial infarction [29].

In our study, only one dosing scheme of VPA was employed. This dose was designed to be relatively low or modest, based on common patient dosages, and was given steadily over days. Serum VPA levels were shown to be below detection levels during the actual experiment (12 hours after last dose), though plasma clearance of this type of drug in pigs is recognised to be rapid. There are no published findings to guide VPA dosing in pigs. Despite these low VPA doses, there was still a HDACi effect on t-PA release, though it is possible that a higher dose could produce a larger effect, as well as also potentially more side-effects. These results therefore shed no light on which aspect of the dosing was most relevant for effect: single dose size and maximal drug exposure, dose interval and drug exposure over time, whether short or long drug exposure is needed, or some other aspect. In this study design, we did not measure degree of general DNA histone acetylation related to VPA treatment, since this has been well demonstrated in other reports, both *in vitro* and *in vivo* [23], [30], [31]. In this porcine model, the animals were young and healthy. It is not possible to generalize from these results concerning HDACi treatment responses in clinical settings where there is thrombotic tendency or deficient t-PA expression. The animals in the non-treatment group had presumably normal constitutive t-PA levels and t-PA release during provocation.

Concerning study design, a standardized ischemic provocation was selected to provide a consistent sub-maximal stimulation for coronary t-PA release [7]. It has been previously shown that repetitive stress stimulation of t-PA can lead to decline in acute t-PA release capacity of the coronary vascular tree, though recovery is rapid [3], [32]. In our model, the surgical preparation involved a sternotomy and pericardiotomy, followed by a long period of rest before the ischemic provocation. An earlier report has shown that acute stimulus in the form of a surgical action (sternotomy) in a porcine model induces acute coronary release of t-PA [33], also with rapid recovery, and this is in line with our results for a reproducible model for stimulation of trans-coronary t-PA release.

These results have demonstrated a proof of concept of HDACi positive effect on t-PA expression in an in vivo setting. Timely acute local release of t-PA is one of many relevant factors in normal thrombosis and fibrinolysis function. These findings from an animal model demonstrate that HDACi intervention *in vivo* have promise for fibrinolytic therapy, though details concerning dose-effect, optimal treatment intervals, and duration of effect were not tested here. Similar findings need to be reproduced in a clinical material, where effects in healthy and chronically ill subjects need to be tested.
